# Study on the interaction between different pathogens of Hand, foot and mouth disease in five regions of China

**DOI:** 10.3389/fpubh.2022.970880

**Published:** 2022-09-27

**Authors:** Zimei Yang, Jia Rui, Li Qi, Wenjing Ye, Yan Niu, Kaiwei Luo, Bin Deng, Shi Zhang, Shanshan Yu, Chan Liu, Peihua Li, Rui Wang, Hongjie Wei, Hesong Zhang, Lijin Huang, Simiao Zuo, Lexin Zhang, Shurui Zhang, Shiting Yang, Yichao Guo, Qinglong Zhao, Shenggen Wu, Qin Li, Yong Chen, Tianmu Chen

**Affiliations:** ^1^State Key Laboratory of Molecular Vaccinology and Molecular Diagnostics, School of Public Health, Xiamen University, Xiamen, Fujian, China; ^2^Chongqing Municipal Center for Disease Control and Prevention, Chongqing, China; ^3^Fujian Center for Disease Control and Prevention, Fuzhou, Fujian, China; ^4^Chinese Center for Disease Control and Prevention, Beijing, China; ^5^Hunan Center for Disease Control and Prevention, Changsha, Hunan, China; ^6^Jilin Center for Disease Control and Prevention, Changchun, Jilin, China; ^7^Department of Stomatology, School of Medicine, Xiamen University, Xiamen, China

**Keywords:** HFMD, interaction, transmissibility, pathogens, mathematical model

## Abstract

**Objectives:**

This study aims to explore the interaction of different pathogens in Hand, foot and mouth disease (HFMD) by using a mathematical epidemiological model and the reported data in five regions of China.

**Methods:**

A cross-regional dataset of reported HFMD cases was built from four provinces (Fujian Province, Jiangsu province, Hunan Province, and Jilin Province) and one municipality (Chongqing Municipality) in China. The subtypes of the pathogens of HFMD, including Coxsackievirus A16 (CV-A16), enteroviruses A71 (EV-A71), and other enteroviruses (Others), were included in the data. A mathematical model was developed to fit the data. The effective reproduction number (*R*_*eff*_) was calculated to quantify the transmissibility of the pathogens.

**Results:**

In total, 3,336,482 HFMD cases were collected in the five regions. In Fujian Province, the *R*_*eff*_ between CV-A16 and EV-A71&CV-A16, and between CV-A16 and CV-A16&Others showed statistically significant differences (*P* < 0.05). In Jiangsu Province, there was a significant difference in *R*_*eff*_ (*P* < 0.05) between the CV-A16 and Total. In Hunan Province, the *R*_*eff*_ between CV-A16 and EV-A71&CV-A16, between CV-A16 and Total were significant (*P* < 0.05). In Chongqing Municipality, we found significant differences of the *R*_*eff*_ (*P* < 0.05) between CV-A16 and CV-A16&Others, and between Others and CV-A16&Others. In Jilin Province, significant differences of the *R*_*eff*_ (*P* < 0.05) were found between EV-A71 and Total, and between Others and Total.

**Conclusion:**

The major pathogens of HFMD have changed annually, and the incidence of HFMD caused by others and CV-A16 has surpassed that of EV-A71 in recent years. Cross-regional differences were observed in the interactions between the pathogens.

## Introduction

Hand, foot and mouth disease (HFMD) is a common infectious disease in children that is caused by a variety of enteroviruses. HFMD was first reported in New Zealand on 19 April, 1957 (1), with eight cases noted in children, and has since emerged in other parts of the world. The HFMD was first reported in China in 1974 (2) and formally included in the management of category C statutory infectious diseases in May 2008. Multiple pathogens of enteroviruses can cause HFMD, and the most common causative agents are Coxsackievirus A16 (CV-A16) and Enteroviruses A71 (EV-A71) (3, 4). CV-A16 was first isolated in South Africa in 1955 (5), and can be divided into genotypes A, B1, and B2. EV-A71 was first reported in California in 1969 (6), and usually causes severe cases in large outbreaks.

Currently, the incidence rate of HFMD is on a highly increasing epidemic trend, and the report and fatality rates rank first among Class C infectious diseases throughout the year (7). At the same time, it imposes a heavy disease burden on the patient's family and the socio-medical system, especially in severe cases of premature death. Losses and socioeconomic burden due to premature death increased 2.0 times to 85.104 million yuan from 2013 to 2015 (8). Studies have shown that EV-A71 was the leading pathogen detected in 2009 and 2010, accounting for 63 and 82%, respectively. Since 2011, the proportion of EV-A71 has dropped to 11%; however, the proportion of CV-A16 HFMD has increased to 51%, and the proportion of HFMD caused by other enteroviruses has also risen to 38% (9). To date, other enteroviruses (such as Coxsackievirus A10) have replaced EV-A71 and CV-A16 as the main pathogens in new cases of HFMD in mainland China (10). China launched two inactivated monovalent EV-A71 vaccines in 2016, with other types of viral vaccines still under development (11). The use of the EV-A71 vaccine can provide infants and young children with at least 1 year of protection against moderate and severe diseases of the EV-A71 virus, with an effectivity rate of 97.4% (12). However, it does not provide immunity to other serological viruses. The current National Immunization Program vaccine in China do not include the monovalent EV-A71 vaccine, and the vaccine coverage rate for children aged 6 months to 5 years is <10–50% (13). The application of the EV-A71 vaccine in China may change the trend of the HFMD virus classification. Exploring the interactions between multiple viruses and their regular patterns can provide more accurate decision-making suggestions for the prevention and control of HFMD, and vaccination coverage.

There have been many studies on the different pathogens of HFMD, but most of them have used traditional epidemiological methods, mainly to describe the incidence of different pathogens of HFMD and the detection rates of different virus types (7, 14, 15). Several studies have used mathematical models to calculate transmissibility of HFMD, as well as study seasonal epidemiological trends (16–18). These studies focused on HFMD caused by EV-A71 alone or HFMD related to all pathogens, and did not consider the potential impact of specific pathogen infections on the transmissibility of other pathogens. Few studies have used transmission dynamics models to explore the transmissibility of different subtypes of HFMD and their interactions. Previous studies have used the susceptible–infectious–recovered (SIR) model to study different pathogens to explore the transmission dynamics of HFMD by city (19), and the results showed that there was an interaction between pathogens (CV-A16 and other enterovirus, EV-A71 and other enterovirus). However, other previous studies have shown that the epidemiological characteristics of HFMD and the early warning times of different regions in China are quite different (20–22). The differences in epidemiological characteristics of HFMD among each regions are multiple and may be related to environmental factors, hosts, and pathogen interactions. Some studies have pointed out that meteorological factors such as daily average pressure, daily average relative humidity, daily average temperature were associated with the incidence of HFMD (23). Socioeconomic and population may also have an impact on morbidity of HFMD (24). In addition, differences in the interaction patterns between pathogens need to be considered. We observed interactions between the different types in one city in China, but it is unclear whether there are interactions between pathogens in other regions and whether they follow the same rules of interaction.

In this study, we chose the susceptible–exposed–infectious–asymptomatic–recovered (SEIAR) model with seasonal characteristics and selected a total of 3,336,482 HFMD case data from four provinces and one municipality, which included East China (Fujian Province and Jiangsu Province), the Central of China (Hunan Province), Southwest China (Chongqing Municipality), and Northeastern China (Jilin Province) to conduct a study on the transmission dynamics of different pathogens. This study had a wide range of research areas and a large sample size of research data, which were distributed across several regions of China. The transmissibility of different pathogens and their interactions among different regions were analyzed. We provided countermeasure suggestions for the prevention and control measures of HFMD in various regions of China.

## Methods

### Research design

**Figure 1:**
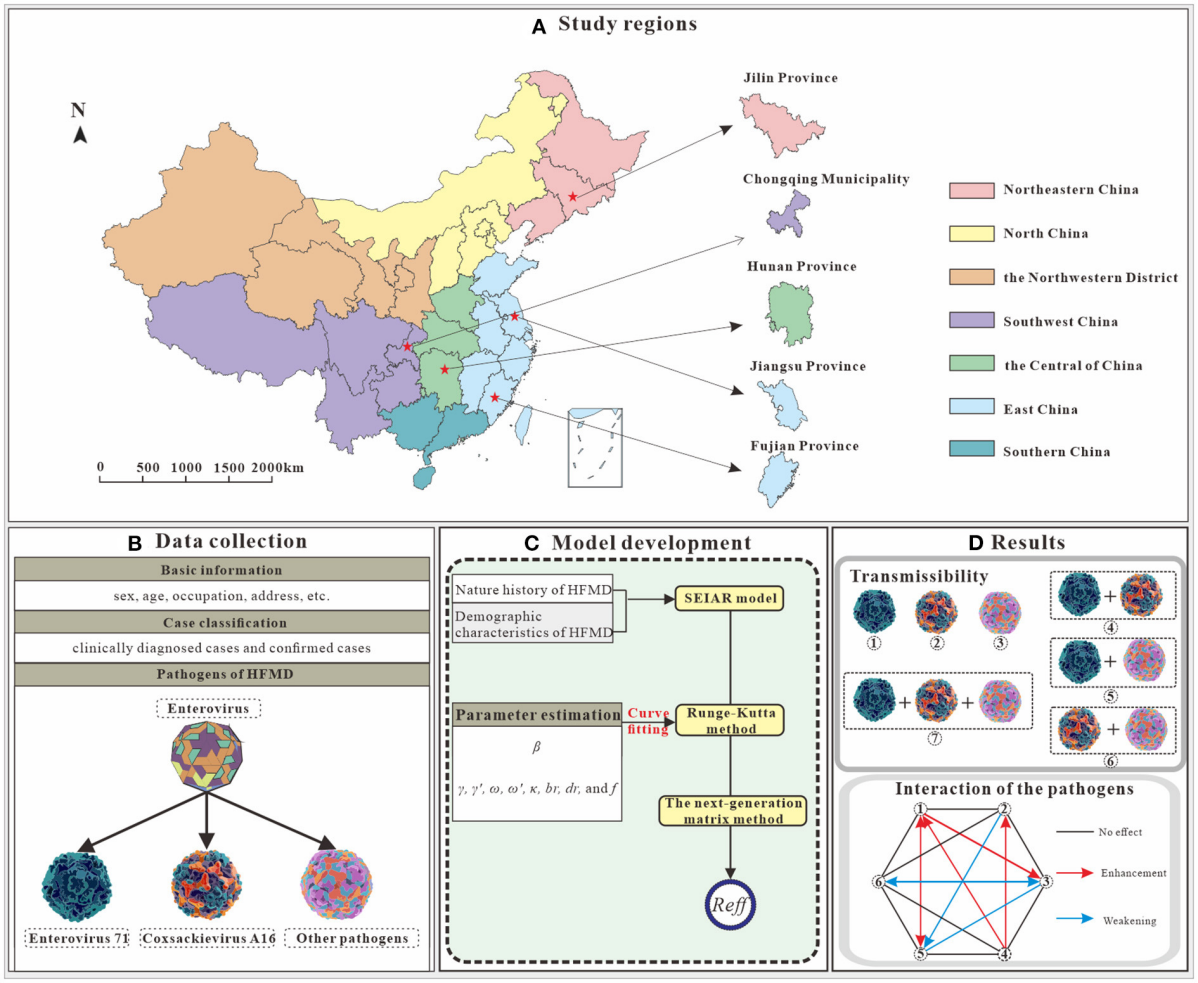
Study design on interaction study of major pathogens of HFMD [**(A)** We selected five study areas from four administrative regions. **(B)** Collection of basic information, HFMD incidence and pathogen data. **(C)** A SEIAR model was constructed based on the natural history of HFMD, and incidence rate of HFMD were fitted. **(D)** Clues to pathogen interactions were obtained by comparing the *R*_*eff*_ in seven scenarios. Illustrations from https://www.dreamstime.com/stock-illustration-enterovirus-which-causes-hand-foot-mouth-disease-hfmd-colorful-background-d-illustration-model-built-using-data-image81638134, EV-A71; https://www.dreamstime.com/stock-illustration-coxsackievirus-virus-which-causes-respiratory-enteric-brain-infections-enterovirus-isolated-white-background-d-image82681501, CV-A16; https://www.dreamstime.com/stock-illustration-enterovirus-d-which-causes-respiratory-infections-children-isolated-white-background-illustration-model-built-using-image81605888, other enterovirus].

Based on the literature review, we selected the SEIAR model, and evaluated viral interactions among different pathogens for the purpose of the study. The research design is illustrated in Figure 1.

### Research objects

Fujian Province, Jiangsu Province, Hunan Province, Chongqing Municipality and Jilin Province were selected as the study areas. The time scale of data sets for each region is as follows: January 2008 to December 2020 in Fujian Province (685,463), January 2015 to December 2019 in Jiangsu Province (642,689), January 2008 to December 2018 in Hunan Province (1,364,315), January 2009 to December 2020 in Chongqing Municipality (476,969), and January 2008 to December 2019 in Jilin Province (167,046). A total of 3,336,482 reported cases of HFMD in the five study regions were used as the research objects.

The study consisted of two main datasets, one included the daily reported number of HFMD cases and deaths in five regions. The second dataset was based on laboratory pathogen examinations. Pathogens (CV-A16, EV-A71, and other enteroviruses) were identified by analysis in the Centers for Disease Control and Prevention (CDC) laboratory using polymerase chain reaction.Both datasets were collected from surveillance data of CDC. Data were collected according to the guidelines for the treatment of HFMD, and the pathogens were classified into three categories (EV-A71, CV-A16, other enterovirus) in the data set. To improve the accuracy of the data, Chinese infectious diseases reporting system conducts regular under-reporting surveys and the data was revised in a timely manner when omissions or false positives were detected.

### Transmission model

In this study, the SEIAR model with seasonal characteristics was selected to fit the daily reported HFMD data in the five study regions. We obtained the transmission rate coefficient (β) by model fitting and further evaluated the transmissibility of the different pathogens. Model construction and parameter estimation methods were obtained from Huang et al. (25). The natural history of the disease in HFMD and the model hypotheses and model framework is shown in the Additional file 1.

### The model equations for the SEIAR model


$$\begin{array}{lll} d/dt\left(S\right) & = & brN-\beta S\left(I+kA\right)-drS\\ d/dt\left(E\right) & = & \beta S\left(I+kA\right)-p\omega E-\left(1-p\right)\omega E-drE\\ d/d\left(I\right) & = & p\omega E-\gamma I-\left(dr+f\right)I\\ d/d\left(A\right) & = & \left(1-p\right)\omega E-\gamma^{\prime }A-drA\\ d/d\left(R\right) & = & \gamma I+\gamma^{\prime }A-drR \end{array}$$

### Estimation of parameters

The definitions and values of the parameters are shown in Table 1.

**Table 1:** Parameter definitions and values.

**Parameters**	**Description**	**Unit**	**Range of value**	**Value**	**Method**
*br*	Birth rate	1	0–1	–	From National Statistical Yearbook
*dr*	Death rate	1	0–1	–	From National Statistical Yearbook
β	Transmission rate coefficient	individual^−1^·day^−1^	0–1	–	Curve fitting
*k*	Relative transmissibility rate of asymptomatic to symptomatic individuals	1	0–1	1	–
*p*	Proportion of the symptomatic	1	0–1	0.4423	References (7, 26, 27)
ω	Incubation relative rate	day^−1^	0–1	0.2	References (26, 28)
γ	Recovery rate of the infectious	day^−1^	0–1	0.07143	References (17, 29)
*γ'*	Recovery rate of the asymptomatic	day^−1^	0–1	0.04762	References (7, 28, 30)
*f*	Fatality rate of HFMD cases	1	0–1	0.0003	References (31–33)

### The seasonality of the transmission

According to the SEAIR model, the seasonality of HFMD should be dynamic and centered on β and the trigonometric functions was adopted for the seasonality in our study with the following equations:

$\beta =\beta_{0}\left[1+\sin\left(\frac{2\pi \left(t+\alpha \right)}{T}\right)\right]$ In the equation, β_0_, *t*, α and *T* refer to the baseline of the transmission rate, time, a constant which adjusts the position of time, and the time span of the season cycle, respectively.

### Assessment of transmissibility

The transmissibility of an infectious disease is usually assessed quantitatively using the basic reproduction number (*R*_0_), which is defined as the number of new cases expected to be generated during the communication period when one case is imported into a susceptible population (34). However, *R*_0_ quantifies the transmissibility of a disease in an ideal state. In cases where the population is not fully susceptible or is under intervention, the transmissibility of an infectious disease should be expressed as the effective reproduction number (*R*_*eff*_). The *R*_*eff*_ was defined as the average number of actual secondary cases for a single case at any time during the epidemic period (35). The formula for calculating the value of *R*_*eff*_ for the SEIAR model is as follows:


$$\begin{array}{l} R_{eff}=\beta S\left(\frac{1-p}{\gamma +f}+\frac{kp}{\gamma }\right) \end{array}$$

### Interaction of different pathogens

Seven scenarios were set up in this study based on serological surveillance data: (1) EV-A71 individual transmission (EV-A71), (2) CV-A16 individual transmission (CV-A16), (3) other enterovirus (others), (4) EV-A71 and CV-A16 co-transmission (EV-A71&CV-A16), (5) EV-A71 and other enterovirus co-transmission (EV-A71&others), (6) CV-A16 and other enterovirus co-transmission (CV-A16&others), (7) All-pathogen co-transmission (Total). The above seven scenarios are not real world scenarios, they are simulated scenarios based on laboratory data. The number of daily incidences for different scenarios = Average monthly composition ratio ^*^ daily incidence of HFMD. The *R*_*eff*_ was estimated for each city and district in the five study areas to quantify the transmissibility of each subgroup.

The interaction pattern between pathogens can be classified as follows (Take pathogen A and pathogen B for example):

1. *R*_*eff*_-A < *R*_*eff*_-A+B; *R*_*eff*_-B = *R*_*eff*_-A+B: Pathogen B and pathogen A have a higher transmissibility when present together than when pathogen A was present alone, and did not differ from pathogen B when it was present alone.
2. *R*_*eff*_-A > *R*_*eff*_-A+B; *R*_*eff*_-B = *R*_*eff*_-A+B: Pathogen B and pathogen A have a lower transmissibility when present together than when pathogen A was present alone, and did not differ from pathogen B when it was present alone.
3. *R*_*eff*_-A < *R*_*eff*_-A+B; *R*_*eff*_-B < *R*_*eff*_-A+B: Pathogen B and pathogen A have a higher transmissibility when present together than when pathogen A or pathogen B was present alone.
4. *R*_*eff*_-A > *R*_*eff*_-A+B; *R*_*eff*_-B < *R*_*eff*_-A+B: Pathogen B and pathogen A have a lower transmissibility when present together than when pathogen A or pathogen B was present alone.

### Statistical analysis

Model fitting was performed using Berkeley Madonna software 8.3.18 (developed by Robert Macey and George Oster of the University of California at Berkeley) to analyse the daily incidence rate of HFMD. The fourth-order Runge–Kutta method, with tolerance set at 0.001, was used to perform curve fitting. When the curve is fitted, Berkeley Madonna shows the root mean square deviation between the data and the best run so far. The optimal results were tested for goodness of fit with the actual data, and quantified using the coefficient of determination (*R*^2^). SPSS 13.0 (IBM Corp, Armonk, NY, USA) was employed to calculate the *R*^2^. The *R*_*eff*_ averages for the different scenarios were compared using analysis of variance, with *P* < 0.05 being a statistically significant difference.

## Results

### Composition ratio of different pathogens of HFMD

**Figure 2:**
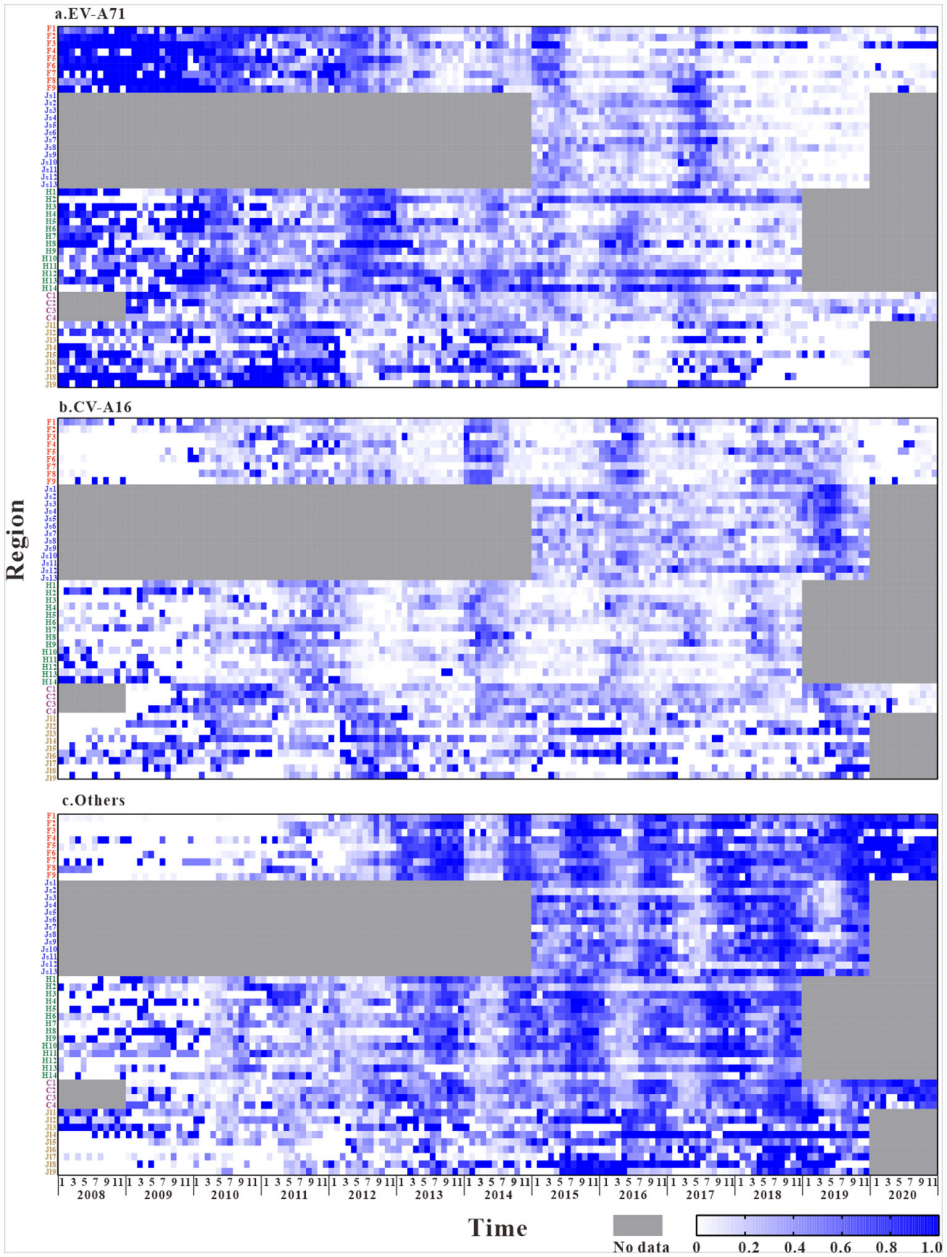
The composition ratio characteristics of different pathogens in the five regions (The composition ratio of EV-A71 = Number of EV-A71 tests per month / Total number of laboratory tests per month, The composition ratio of CV-A16 = Number of CV-A16 tests per month / Total number of laboratory tests per month, The composition ratio of Others = Number of Others tests per month / Total number of laboratory tests per month. F1, Fuzhou City; F2, Xiamen City; F3, Putian City; F4, Sanming City; F5, Quanzhou City; F6, Zhangzhou City; F7, Nanping City; F8, Longyan City; F9, Ningde City; Js1, Nanjing City; Js2, Wuxi City; Js3, Xuzhou City; Js4, Changzhou City; Js5, Suzhou City; Js6, Nantong City; Js7, Lianyungang City; Js8, Huaian City; Js9, Yancheng City; Js10, Yangzhou City; Js11, Zhenjiang City; Js12, Taizhou City; Js13, Suqian City; H1, Changsha City; H2, Zhuzhou City; H3, Xiangtan City; H4, Hengyang City; H5, Shaoyang City; H6, Yueyang City; H7, Changde City; H8, Zhangjiajie City; H9, Yiyang City; H10, Chenzhou City; H11, Yongzhou City; H12, Huaihua City; H13, Loudi City; H14, Xiangxi Prefecture; C1, The central urban area of Chongqing; C2, The new area of Chongqing city proper; C3, The city cluster of three gorges reservoir area in northeast Chongqing; C4, The city cluster of Wuling mountain area in southeast Chongqing; Jl1, Changchun City; Jl2, Jilin City; Jl3, Siping City; Jl4, Liaoyuan City; Jl5, Tonghua City; Jl6, Baishan City; Jl7, Songyuan City; Jl8, Baicheng City; Jl9, Yanbian Prefecture).

A total of 3,336,482 reported cases of HFMD in the five study regions were used as the research objects. As the Figure 2 shows the composition ratio of EV-A71 have a significant decrease after 2018 in all regions. And the composition ratio of other enteroviruses has increased in recent years. However, the composition ratio of CV-A16 have not changed.

The laboratory results of HFMD in different regions (Figure 2) show that, during the years of this study, HFMD cases in the southeastern coastal region of East China were mainly caused by EV-A71 and other enterovirus, while fewer were caused by CV-A16. The disease was mostly induced by EV-A71 in the first 5 years, and the composition ratio of other enterovirus increased significantly after 2016. Similarly, the Central of China had a larger composition ratio of EV-A71 and other enterovirus. In Southwest China and the Yellow Sea region of East China, HFMD is mainly caused by other enterovirus. The disease-causing pathogen of HFMD in Northeastern China is mainly CV-A16.

The composition ratios of the different pathogens in the five regions showed certain trends over time. In general, the composition ratio of CV-A16 did not change significantly and was relatively stable, with only a small increase in the Yellow Sea coastal region of East China after 2018. The composition of EV-A71 showed a clear trend over time. At the national level, a relatively large proportion of the composition of EV-A71 was shown until 2016, with a trend of decreasing year by year thereafter.In contrast, the composition ratio of other enterovirus showed an opposite trend over time, with a lower composition of <40% in the initial years and a gradual increase in the proportion since 2015.

### Transmissibility of the different pathogens of HFMD

**Figure 3:**
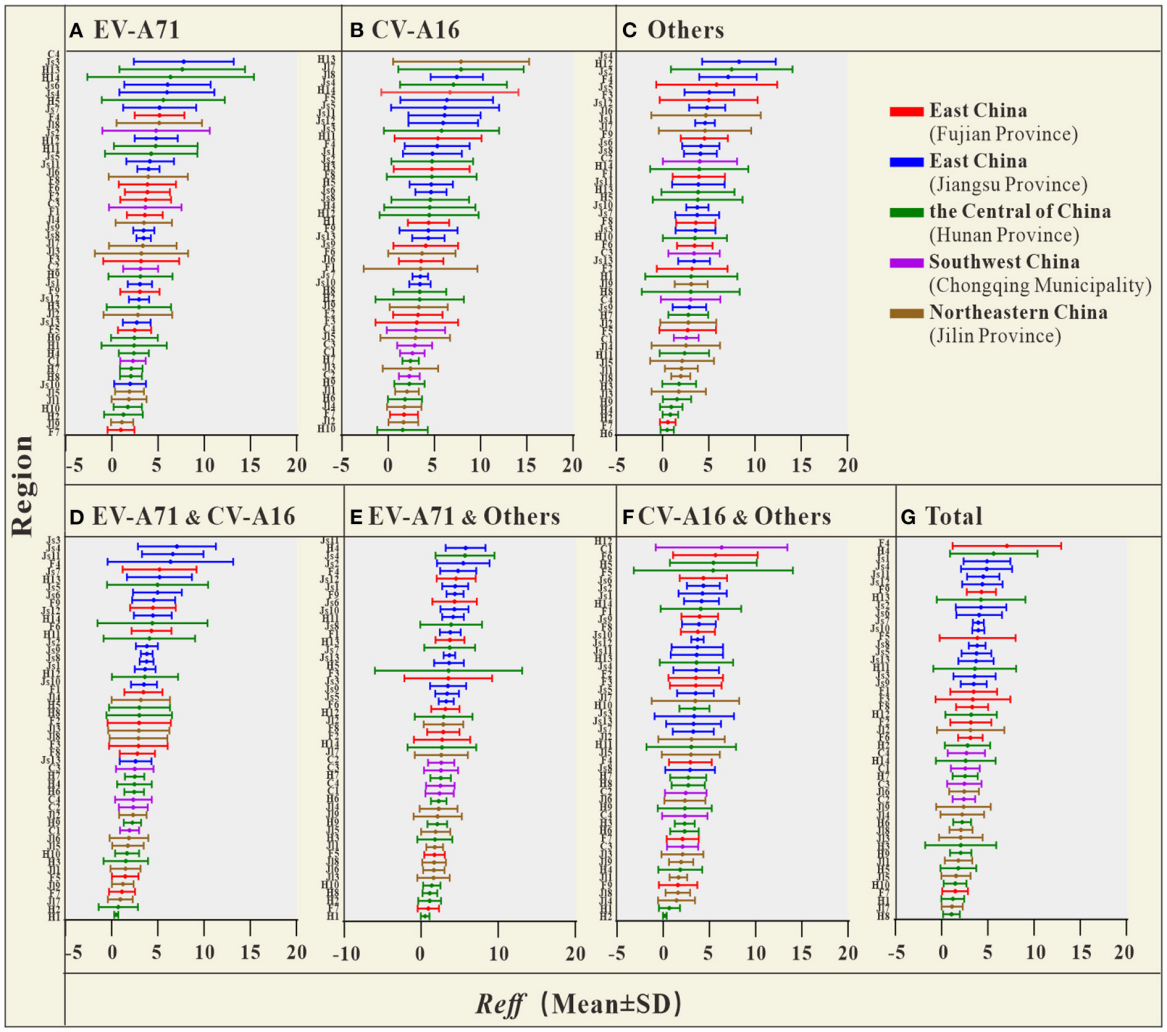
The *R*_*eff*_ of HFMD in each city, autonomous prefecture, and district [**(A)** *R*_*eff*_ average of EV-A71; **(B)** *R*_*eff*_ average of CV-A16; **(C)** *R*_*eff*_ average of Others; **(D)** *R*_*eff*_ average of EV-A71&CV-A16; **(E)** *R*_*eff*_ average of EV-A71&Others; **(F)** *R*_*eff*_ average of CV-A16&Others; **(G)** *R*_*eff*_ average of Total] (F1, Fuzhou City; F2, Xiamen City; F3, Putian City; F4, Sanming City; F5, Quanzhou City; F6, Zhangzhou City; F7, Nanping City; F8, Longyan City; F9, Ningde City; Js1, Nanjing City; Js2, Wuxi City; Js3, Xuzhou City; Js4, Changzhou City; Js5, Suzhou City; Js6, Nantong City; Js7, Lianyungang City; Js8, Huaian City; Js9, Yancheng City; Js10, Yangzhou City; Js11, Zhenjiang City; Js12, Taizhou City; Js13, Suqian City; H1, Changsha City; H2, Zhuzhou City; H3, Xiangtan City; H4, Hengyang City; H5, Shaoyang City; H6, Yueyang City; H7, Changde City; H8, Zhangjiajie City; H9, Yiyang City; H10, Chenzhou City; H11, Yongzhou City; H12, Huaihua City; H13, Loudi City; H14, Xiangxi Prefecture; C1, The central urban area of Chongqing; C2, The new area of Chongqing city proper; C3, The city cluster of three gorges reservoir area in northeast Chongqing; C4, The city cluster of Wuling mountain area in southeast Chongqing; Jl1, Changchun City; Jl2, Jilin City; Jl3, Siping City; Jl4, Liaoyuan City; Jl5, Tonghua City; Jl6, Baishan City; Jl7, Songyuan City; Jl8, Baicheng City; Jl9, Yanbian Prefecture).

The coefficient of determination (*R*^2^) represents the goodness-of-fit of the model used, which is shown in Additional file 2 with a well-fitted effect (*R*^2^ in most cities was > 0.5, and *P* < 0.05). The *R*_*eff*_ values calculated for different scenarios are shown in Figure 3. Overall, the *R*_*eff*_ values of HFMD in Jiangsu Province (in the Yellow Sea region of East China) were higher than those in the Central of China, Southwest China and Northeastern China.

The results of the study in Table 2 show that, the *R*_*eff*_ of HFMD differed significantly between regions. The difference in *R*_*eff*_ values between the two provinces in East China was smaller (0.395), but this difference was not statistically significant (*P* > 0.05). The differences in *R*_*eff*_ between the two regions of the Central of China, Southwest China and Northeastern China were not statistically significant (*P* > 0.05). In contrast, *R*_*eff*_ was significantly different between each of the two provinces in East China and the other three regions (*P* < 0.05).

**Table 2:** Comparison of the transmissibility of HFMD in the study regions.

**Compare regions**		**Difference of *R_*eff*_***	***P-*value**
**Region A**	**Region B**	**(A-B)^a^**	
East China (Fujian Province) vs.	East China (Jiangsu Province)	−0.395	0.574
	Northeastern China (Jilin Province)	1.621	0.000^*^
	the Central of China (Hunan Province)	1.096	0.000^*^
	Southwest China (Chongqing)	1.198	0.000^*^
East China (Jiangsu Province) vs.	Northeastern China (Jilin Province)	2.016	0.000^*^
	the Central of China (Hunan Province)	1.492	0.000^*^
	East China (Fujian Province)	0.395	0.574
	Southwest China (Chongqing)	1.593	0.000^*^
The Central of China (Hunan Province) vs.	East China (Jiangsu Province)	−1.492	0.000^*^
	Northeastern China (Jilin Province)	0.525	0.202
	East China (Fujian Province)	−1.096	0.000^*^
	Southwest China (Chongqing)	0.101	0.994
Southwest China (Chongqing) vs.	East China (Jiangsu Province)	−1.593	0.000^*^
	Northeastern China (Jilin Province)	0.424	0.426
	the Central of China (Hunan Province)	−0.101	0.994
	East China (Fujian Province)	−1.198	0.000^*^
Northeastern China (Jilin Province) vs.	East China (Jiangsu Province)	−2.016	0.000^*^
	the Central of China (Hunan Province)	−0.525	0.202
	East China (Fujian Province)	−1.621	0.000^*^
	Southwest China (Chongqing)	−0.424	0.426

* The statistical methods is Analysis of Variance. *P* < 0.05 indicates the difference of *R*_*eff*_ between two regions was statistically significant.

a Difference of *R*_*eff*_ is the *R*_*eff*_ mean of region A minus the *R*_*eff*_ mean of region B.

### Interaction of different pathogens

**Figure 4:**
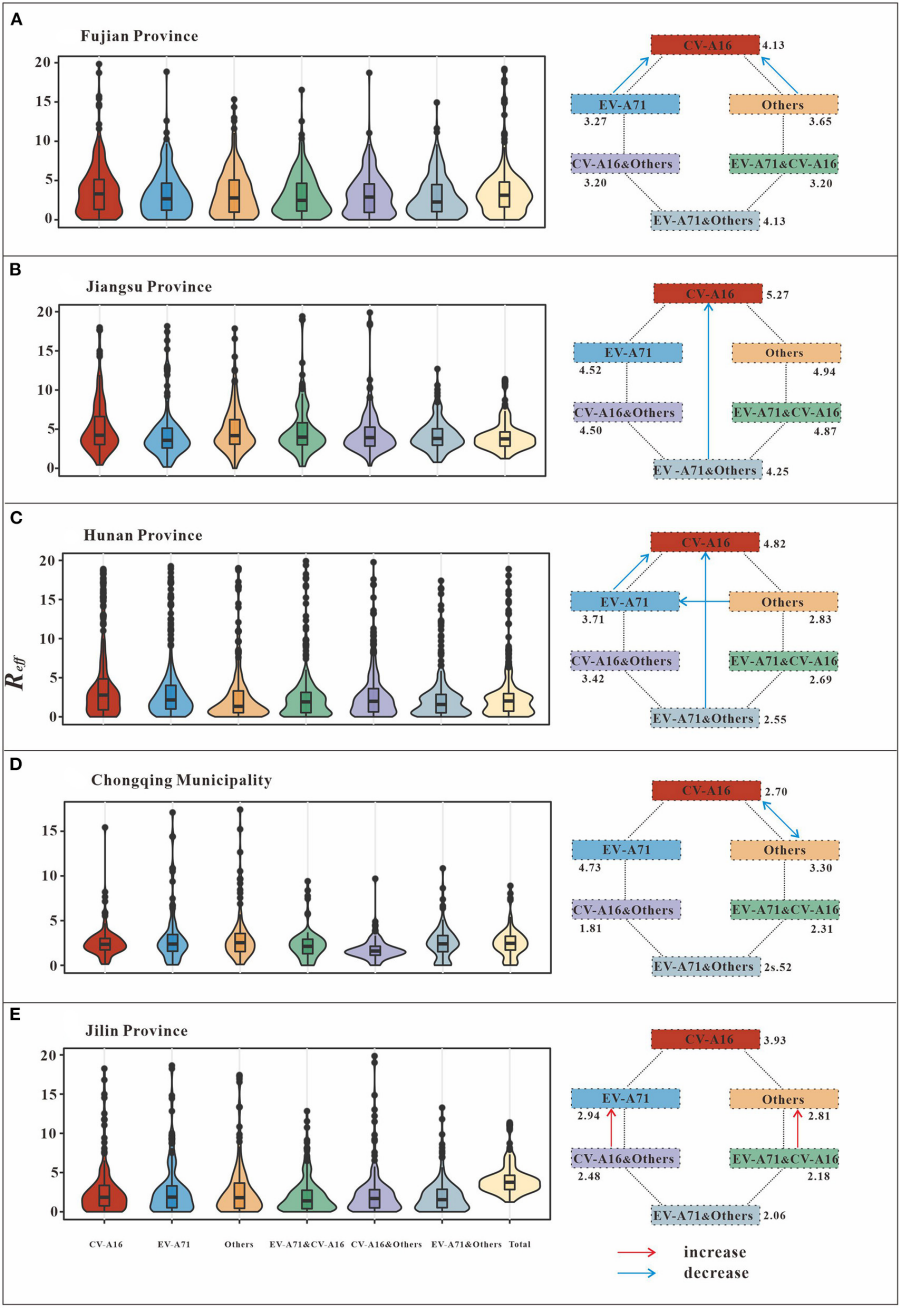
Schematic diagram of the interaction among different pathogens of HFMD. **(A)** (Fujian Province) indicates EV-A71 has negative inhibition on CV-A16, while others have negative inhibition on CV-A16. **(B)** (Jiangsu Province) shows that EV-A71 and others are present together to produce negative inhibition of CV-A16. **(C)** (Hunan Province) indicates EV-A71 has negative inhibition on CV-A16. There is negative inhibition of CV-A16 when EV-A71 and others are transmitted simultaneously. In addition to this, others have negative inhibition on EV-A71. **(D)** (Chongqing Municipality) indicates a bidirectional negative inhibition between CV-A16 and other. **(E)** (Jilin Province) indicates that when all-pathogen co-transmission, EV-A71, and other enterovirus get some increase in transmissibility.

In this study, we analyzed the interaction between pathogens by comparing the *R*_*eff*_ values of different scenarios (Figure 4; Table 3). The results from Fujian Province, the southeastern coastal region of East China, showed that the differences in *R*_*eff*_between CV-A16 (mean of *R*_*eff*_ = 4.13) and EV-A71&CV-A16 (mean of *R*_*eff*_ = 3.20), as well as that between CV-A16 (Mean of *R*_*eff*_ = 4.13) and CV-A16&others (mean of *R*_*eff*_ = 3.20) scenarios was statistically significant (*P* < 0.05). This significant difference was not observed in the comparison between the other scenarios (*P* > 0.05). In Jiangsu Province, the Yellow Sea region of East China, there was a significant difference in *R*_*eff*_ (*P* < 0.05) between the CV-A16 (mean of *R*_*eff*_ = 5.27) and total (mean of *R*_*eff*_ = 4.13) scenarios.

**Table 3:** Interaction pattern of different pathogens of HFMD.

**Region**	**Scenarios**		**Difference of *R_*eff*_***	***P-*value**
	**A**	**B**	**(A-B)^a^**	
East China (Fujian Province)	CV-A16	EV-A71&CV-A16	0.931	0.039^*^
	CV-A16	CV-A16&Others	0.929	0.044^*^
	CV-A16	Total	0.390	0.898
	EV-A71	EV-A71&CV-A16	0.075	1.000
	EV-A71	EV-A71&Others	0.226	0.969
	EV-A71	Total	−0.466	0.594
	Others	CV-A16&Others	0.451	0.705
	Others	EV-A71&Others	0.605	0.356
	Others	Total	−0.088	1.000
East China (Jiangsu Province)	CV-A16	EV-A71&CV-A16	0.399	0.947
	CV-A16	CV-A16&Others	0.773	0.361
	CV-A16	Total	1.140	0.009^*^
	EV-A71	CV-A16&EV-A71	−0.350	0.981
	EV-A71	EV-A71&Others	0.274	0.989
	EV-A71	Total	0.390	0.934
	Others	CV-A16&Others	0.439	0.860
	Others	EV-A71&Others	0.689	0.237
	Others	Total	0.806	0.088
The Central of China (Hunan Province)	CV-A16	EV-A71&CV-A16	2.122	0.001^*^
	CV-A16	CV-A16&Others	1.394	0.435
	CV-A16	Total	2.175	0.001^*^
	EV-A71	EV-A71&CV-A16	1.015	0.050^*^
	EV-A71	EV-A71&Others	1.160	0.017^*^
	EV-A71	Total	1.068	0.020^*^
	Others	CV-A16&Others	−0.594	0.941
	Others	EV-A71&Others	0.279	0.978
	Others	Total	0.187	0.996
Southwest China (Chongqing)	CV-A16	EV-A71&CV-A16	0.398	0.777
	CV-A16	CV-A16&Others	0.898	0.008^*^
	CV-A16	Total	0.168	0.996
	EV-A71	EV-A71&CV-A16	2.422	0.531
	EV-A71	EV-A71&Others	2.215	0.637
	EV-A71	Total	2.192	0.646
	Others	CV-A16&Others	1.491	0.000^*^
	Others	EV-A71&Others	0.784	0.327
	Others	Total	0.761	0.329
Northeastern China (Jilin Province)	CV-A16	EV-A71&CV-A16	1.757	0.479
	CV-A16	CV-A16&Others	1.453	0.715
	CV-A16	Total	−0.201	1.000
	EV-A71	EV-A71&CV-A16	0.765	0.340
	EV-A71	EV-A71&others	0.885	0.161
	EV-A71	Total	−1.192	0.012^*^
	Others	CV-A16&others	0.331	0.979
	Others	EV-A71&others	0.755	0.295
	Others	Total	−1.323	0.002^*^

* The statistical methods is Analysis of Variance. *P* < 0.05 indicates the difference of *R*_*eff*_ between two scenarios was statistically significant.

a Difference of *R*_*eff*_ is the *R*_*eff*_ mean of scenario A minus the *R*_*eff*_ mean of scenario B.

In the Central of China (Hunan Province), the difference in *R*_*eff*_ between CV-A16 (mean of *R*_*eff*_ = 4.82) and EV-A71&CV-A16 (mean of *R*_*eff*_ = 2.69), as well as between CV-A16 (mean of *R*_*eff*_ = 4.82) and total (mean of *R*_*eff*_ = 2.64) were statistically significant (*P* < 0.05). The differences in *R*_*eff*_ between EV-A71 (mean of *R*_*eff*_ = 3.71) and EV-A71&CV-A16 (mean of *R*_*eff*_ = 2.69), EV-A71 (mean of *R*_*eff*_ = 3.71) and EV-A71&others (mean of *R*_*eff*_ = 2.55), EV-A71 (mean of *R*_*eff*_ = 3.71) and total (mean of *R*_*eff*_ = 2.64) were statistically significant (*P* < 0.05). However, none of the differences among the other scenarios were statistically significant (*P* > 0.05).

Chongqing Municipality, which is located in Southwest China, found statistically significant differences in the *R*_*eff*_ between CV-A16 (Mean of *R*_*eff*_ = 2.71) and CV-A16 & others (Mean of *R*_*eff*_ = 1.81), as well as between others (Mean of *R*_*eff*_ = 3.30) and CV-A16 & others (Mean of *R*_*eff*_ = 1.81) (*P* < 0.05).

On the other hand, in Jilin Province, Northeastern China, statistically significant (*P* < 0.05) differences in *R*_*eff*_ were found between EV-A71 (mean of *R*_*eff*_ = 2.94) and total (mean of *R*_*eff*_ = 4.13), as well as between others (mean of *R*_*eff*_ = 2.81) and total (mean of *R*_*eff*_ = 4.13).

## Discussion

Multiple pathogens of enterovirus can cause HFMD, and are co-transmitted in the population. Previously, in our group's study on different pathogens of HFMD in Changsha City, the reproduction number was used to measure the transmissibility of different pathogens and their interactions (19); however, it was only analyzed for one city in the Central of China. In previous studies, high prevalence of HFMD was found in southwestern and central as well as eastern China (36), differences between regions may be due to different geographical environments (37). The geographical distribution of China varies widely among different regions, with the eastern region dominated by plains, the southeastern coastal cities with higher temperatures, and the southwestern region dominated by mountainous regions with a subtropical monsoon climate, factors that may favor the outbreak of HFMD (38). In this study, four provinces and one municipality were selected to further exploration of the transmissibility and interaction of different pathogens on a larger scale, including analyse the possible geographical differences. This study was the first to examine the transmission dynamics of different subtypes of HFMD at the provincial level, with a wide study area covering four of the seven administrative regions in China, namely the East China, the Central of China, Southwest China, and Northeastern China. In this study, we analyzed the transmissibility of sub-pathogens based on reliable laboratory results and explored possible interactions between different pathogens of enteroviruses.

The study showed that the predominant pathogen causing the occurrence of severe cases of HFMD in China was overall dominated by EV-A71, but after 2013 and 2015 the percentage of EV-A71 showed a decreasing trend, and in 2018 other enteroviruses became the dominant pathogen (36). In addition, another study showed that during 2008–2016 in China, most EV-A71 was endemic in the eastern, northern, central, and southwestern regions, most CV-A16 was endemic in eastern, southern, and northern China, and other viruses (e.g., CV-A6) were scattered in various regions (39). In this study, a significant trend in the composition ratio of different pathogens over time was observed in all the five study areas. After 2016, there was a significant decrease in the composition ratio of EV-A71 and an increase in the composition ratio of HFMD caused by other enterovirus. This may be because the monovalent EV-A71 virus vaccine was launched in 2016, indicating that the population acquired immunity against EV-A71 with the administration of the vaccine (40). However, other pathogens still have a high transmission rate, which are even higher than that at other time periods.

In China, EV-A71 vaccine is a national class II vaccine, not a mandatory vaccination, and requires self-payment. The optimal age for EV-A71 vaccination is from 6 months to 71 months of age, and full vaccination is encouraged to be completed before the infant reaches 12 months of age (41). The current coverage of EV-A71 vaccine in China is not very high as the previous researches showed that the vaccination coverage rate in Guangdong Province was rose from 3.82 to 10.07% in 2016 and 2017 (42), and the EV71 vaccination rate in Ningbo City was 24.05% among 716,178 children born from 2012 to 2018, with a timely vaccination rate of only 8.61% (43). In Kunming, a central city in southwest China, about 19.16% of children were vaccinated in 2018. Compared to 2015–2016, the number of EV-A71 HFMD cases in Kunming City decreased significantly in 2018 (44). Despite the low vaccination coverage, EV-A71 vaccine implementation continues to have a significant impact on the epidemiological trend of EV-A71 HFMD. In the previous study, we found a significant decrease in the proportion of EV-A71 infections after 2018 in our study of Nanchang City. Correlation analysis showed that EV-A71 vaccination was negatively associated with the incidence of EV-A71 infection (45). Some clinical trials have shown that the EV-A71 vaccine is not protective against HFMD caused by other pathogens (46). In addition, studies have indicated that when vaccination coverage reaches 80–90%, adequate herd immunity can be generated, creating additional social benefits as well (47, 48). Declining vaccination rates can lead to disease outbreaks and epidemics (49, 50). Improving the coverage and timeliness of EV-A71 vaccination is important for disease prevention and control. Meanwhile, it has been suggested that the development of a multivalent HFMD vaccine may be the best strategy, considering the combination of epidemiological, technical, immunological, and economic challenges associated with HFMD (51). Additionally, there are no pharmaceutical interventions for HFMD. Prevention and control is focused on preventing the spread of the virus in the population. Other HFMD prevention and control measures should be given more attention, such as the establishment of a clean, well-hygienic environment in the population, as well as and the timely implementation of isolation of infected cases.

The SEIAR model fitted well with the reported data and the results had good validity. Based on the comparison of HFMD *R*_*eff*_, we found that the transmissibility of HFMD was closer between the two provinces in East China, as well as between the Central of China, Southwest China, and Northeastern China. In contrast, there was a significant difference in the *R*_*eff*_ values between East China and the other three regions, with the transmissibility of HFMD being higher in East China. The spread of HFMD is closely related to temperature and humidity, and some studies have shown that the incidence rate of HFMD increases with higher average temperatures (52). East China, which has high temperature and humidity, as well as a high population density, may be conducive to the sharp spread of HFMD.

The interaction patterns among the HFMD pathogens differed among the five study regions. In the southeastern coastal region of East China (Fujian Province), CV-A16 has a higher transmissibility when transmitted alone than when transmitted with EV-A71 or other enterovirus together. Similarly, in the Yellow Sea region of eastern China (Jiangsu Province), the transmissibility when CV-A16, EV-A71 and other enterovirus are present together is lower than when CV-A16 is present alone. The interactions between different HFMD pathogens in the Central of China are more complex. When CV-A16 and EV-A71 are present together the transmissibility is lower than when they are each present alone. Also, EV-A71 is more transmissible when present alone than when other enterovirus and EV-A71 are present together. All-pathogen co-transmission conditions have a lower transmissibility than CV-A16 or EV-A71 alone. This result was generally consistent with the interactions between various pathogens in Changsha City during the rising phase of the disease. In Southwest China, CV-A16 and other enterovirus have a much lower transmissibility when present together. Completely different results were observed in Northeastern China. In the case of all-pathogen co-transmission, EV-A71, and other enterovirus increased in transmissibility, whereas CV-A16 did not.

These results show that the interaction of different pathogens varies greatly with geography. There is empirical evidence that infection with a multi-pathogen viral pathogen (e.g., influenza virus) confers transient immunity against other pathogens of the same infection (53). For HFMD, the incidence rate of patients infected with both pathogens is low and there may be a short-term cross-protective effect (54). Although studies have presented evidence for interactions between different HFMD pathogens in terms of transmission dynamics (19), the complexity of these interactions is not known. This may be related to differences in climate (temperature, relative humidity, rainfall, etc.) and cultural and social factors between the regions. The former has a limiting effect on the activity of pathogens, whereas the latter may influence individual lifestyle, hygiene habits, and human contact patterns (55). In conclusion, the interaction between different HFMD pathogens may be highly variable with geography and the mechanisms are complex. Overall, there is a dilution effect of transmission when multiple subtypes of pathogens are transmitted simultaneously, compared to the circulation of just a single predominant subtype. Further studies are required to determine relevant issues.

Of notes, there are several limitations in this study. Firstly, due to the limitations of the data source, this study could only explore the relationship between the three subtypes of HFMD (CV-A16, EV-A71 and other enterovirus). Other enterovirus were not further classified for analysis, which may have prevented meaningful effects of other subtypes from being observed. Analysis of interactions between more pathogens could be considered in future studies. Secondly, although Chinese infectious diseases reporting system provides some control over underreporting and misreporting of HFMD, the possible underreporting and misreporting can have some impact on the analysis of the results. Thirdly, immunity plays an important role in the prevalence of HFMD, but model was not considered due to the unavailability of accurate vaccination rate data for EV71 vaccine in all study areas, which may have an impact on the results of the study. If accurate vaccination rate data are available in the future, immunological issues can be taken into account in future studies. In conclusion, the major pathogens of HFMD have changed annually, with the incidence of HFMD caused by others and CV-A16 surpassing that of EV-A71 in recent years. More attention should be paid to East China because of the high transmissibility of HFMD. Cross-regional differences were observed in the interactions between the pathogens. In most regions of China (East China, the Central of China, and Southwest China), the transmissibility of HFMD was reduced when multiple subtypes of virus were transmitted simultaneously in the population compared with single virus transmission. In contrast, in Northeastern China, an increase in subtype transmissibility was rarely observed.

## Data availability statement

The datasets presented in this article are not readily available because the data that support the findings of this study are available from Chinese Center for Disease Control and Prevention but restrictions apply to the availability of these data, which were used under license for the current study, and so are not publicly available. Data are however available from the authors upon reasonable request and with permission of Chinese Center for Disease Control and Prevention. Requests to access the datasets should be directed to TC, 13698665@qq.com.

## Author contributions

JR, ZY, TC, and QL made substantial contributions to conception and design. ZY, JR, YN, LQ, and WY collected literature, drafted the manuscript, conceived the experiments, and wrote the manuscript. YN, LQ, WY, QZ, ZY, and JR collected the data. SW, QL, YC, and TC provided technical help and revised it critically for important intellectual content. ZY, ShiZ, HW, HZ, LH, ShuZ, LZ, SiZ, ShanY, and YG conducted the experiments and analyzed the results. ZY, JR, BD, ShanY, ShiY, CL, PL, and HW involved in the visualization of the results. All authors approved the final manuscript and agreed to be accountable for all aspects of the work.

## Funding

This work was supported by the Bill and Melinda Gates Foundation (No: INV-005834), Chongqing Municipal Health Commission (No: 2019GDRC014), Construction of Fujian Provincial Scientific and Technological Innovation Platform (No: 2019Y2001), Provincial Natural Science Foundation of Fujian Province (No: 2013J01268), Public Health Applied Research and Vaccine Preventable Diseases Research Project, Chinese Society of Preventive Medicine (No: 20101801), Science and Technology Plan Project of Hunan Provincial Science and Technology Department (No: 2011FJ3137), Hunan Provincial Natural Science Foundation Joint Project on Science and Health (No: 2019JJ80115), and Chongqing Science and Technology Bureau (CSTC2021jscx-gksb-N0005).

## Conflict of interest

The authors declare that the research was conducted in the absence of any commercial or financial relationships that could be construed as a potential conflict of interest.

## Publisher's note

All claims expressed in this article are solely those of the authors and do not necessarily represent those of their affiliated organizations, or those of the publisher, the editors and the reviewers. Any product that may be evaluated in this article, or claim that may be made by its manufacturer, is not guaranteed or endorsed by the publisher.

## Abbreviations

HFMD: Hand foot and mouth disease
CV-A16: Coxsackievirus A16
EV-A71: Enterovirus A71
CV-B1: Coxsackievirus B1
SEIAR: susceptible–exposed–infectious–asymptomatic–recovered
SEIR: susceptible–exposed–infectious–recovered
SEIQR: susceptible–exposed–infectious–quarantined–recovered
SEIARW: susceptible–exposed–infectious–asymptomatic–recovered–environment
*R*_0_: the basic reproduction number
*R*_*eff*_: the Effective reproduction number.
