# Supplementary material for: Study on the interaction between different pathogens of Hand, foot and mouth disease in five regions of China
Source: Front Public Health. 2022 Sep 27;10:970880. doi: 10.3389/fpubh.2022.970880 (PMC9552780; doi:10.3389/fpubh.2022.970880)
Supplement: Supplementary file 1 [file Data_Sheet_1.PDF]

Additional file 1. The SEIAR model for HFMD

SEIAR model hypotheses

The SEIAR model was constructed to fit the data. Based on the following hypotheses:

(1) Natural population movements (births, deaths) in each province cause a change in the population base. The birth rate is expressed in *br* and the death rate in *dr*.

(2) Susceptible (S) to contact with Infectious (I) conditions, Infectious have a certain degree of infectivity, and transmission rate coefficient is β. If Susceptible is exposed to Asymptomatic (A), the Asymptomatic is also contagious, k times more than the Infectious ($0 < k < 1$), with transmission rate coefficient of $k\beta$. The rate of transmission from Susceptible to Exposure (E) at moment t is $\beta S(I+kA)$.

(3) After an incubation period of disease, a certain proportion of E turns into I. This proportion is p. The rate of progression from E to I at moment t is proportional to the population of incubation period, with a scale factor of $p\omega$. The other part ($1-p$) of E turns into A after an incubation period of the disease. The rate of progression from E to A is proportional to the latency population, with a scale factor of $(1-p)\omega$.

(4) Assume that the time interval between the onset of symptoms and recovery of health for I is $1/\gamma$. Then the number of people who change from I to Recovery (R) at moment t is γI. Let the rate of death from HFMD be f, then the number of cases that die at time t is fI.

(5) Assume that the time interval between onset and recovery of health for A is $1/\gamma'$. Then the number of people who change from A to R at moment t is $\gamma' A$.

The natural history of the disease in HFMD is as follows：

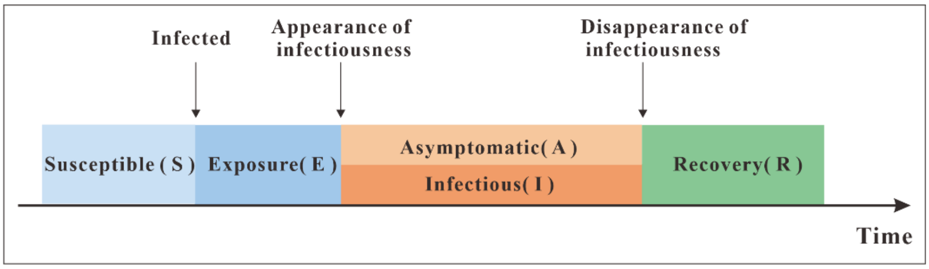
Infected
Appearance of infectiousness
Disappearance of infectiousness
Susceptible (S)
Exposure(E)
Asymptomatic(A)
Infectious(I)
Recovery(R)
Time
