# Supplementary material for: Study on the interaction between different pathogens of Hand, foot and mouth disease in five regions of China
Source: Front Public Health. 2022 Sep 27;10:970880. doi: 10.3389/fpubh.2022.970880 (PMC9552780; doi:10.3389/fpubh.2022.970880)
Supplement: Supplementary file 2 [file Data_Sheet_2.PDF]

Additional file 2 The coefficient of determination (R^2) for different scenarios in the five study regions

	EV-A71	CV-A16	Others	EV-A71& CV-A16	EV-A71+ Others	CV-A16& Others	Total
**East China**							
**(Fujian Province)**							
Fuzhou City	0.444	0.220	0.682	0.590	0.327	0.393	0.591
Xiamen City	0.343	0.662	0.536	0.463	0.332	0.694	0.528
Putian City	0.693	0.753	0.549	0.653	0.539	0.450	0.520
Sanming City	0.585	0.697	0.558	0.520	0.511	0.611	0.548
Quanzhou City	0.710	0.765	0.729	0.738	0.700	0.627	0.746
Zhangzhou City	0.530	0.524	0.570	0.519	0.487	0.561	0.550
Nanping City	0.553	0.625	0.646	0.582	0.617	0.601	0.563
Longyan City	0.626	0.352	0.441	0.443	0.475	0.443	0.443
Ningde City	0.557	0.822	0.619	0.443	0.572	0.724	0.571
**East China**							
**(Jiangsu Provicne)**							
Nanjing City	0.738	0.840	0.692	0.773	0.708	0.712	0.689
Wuxi City	0.722	0.763	0.725	0.755	0.740	0.749	0.760
Xuzhou City	0.426	0.405	0.348	0.544	0.396	0.453	0.354
Changzhou City	0.817	0.687	0.508	0.765	0.586	0.765	0.633
Suzhou City	0.869	0.815	0.770	0.842	0.690	0.707	0.769

Nantong City	0.685	0.734	0.858	0.792	0.835	0.839	0.845
Lianyungang City	0.507	0.720	0.848	0.588	0.616	0.738	0.630
Huaian City	0.784	0.685	0.599	0.615	0.303	0.426	0.430
Yancheng City	0.770	0.623	0.423	0.714	0.440	0.327	0.451
Yangzhou City	0.795	0.798	0.628	0.839	0.695	0.703	0.684
Zhenjiang City	0.730	0.635	0.634	0.706	0.713	0.647	0.698
Taizhou City	0.647	0.810	0.485	0.781	0.541	0.869	0.852
Suqian City	0.604	0.758	0.549	0.419	0.549	0.435	0.603
**the Central of China**							
**(Hunan Province)**							
Changsha City	0.535	0.571	0.518	0.603	0.622	0.602	0.630
Zhuzhou City	0.741	0.652	0.675	0.705	0.707	0.653	0.586
Xiangtan City	0.702	0.691	0.788	0.735	0.724	0.694	0.720
Hengyang City	0.776	0.719	0.664	0.734	0.694	0.546	0.692
Shaoyang City	0.395	0.653	0.705	0.675	0.666	0.493	0.665
Yueyang City	0.503	0.711	0.566	0.668	0.595	0.525	0.624
Changde City	0.502	0.592	0.516	0.705	0.612	0.652	0.612
Zhangjiajie City	0.650	0.577	0.668	0.656	0.676	0.706	0.676
Yiyang City	0.509	0.620	0.676	0.669	0.661	0.574	0.661
Chenzhou City	0.723	0.595	0.792	0.650	0.610	0.694	0.650
Yongzhou City	0.593	0.595	0.500	0.595	0.510	0.698	0.603

Huaihua City	0.738	0.681	0.526	0.613	0.647	0.637	0.665
Loudi City	0.793	0.628	0.755	0.582	0.730	0.793	0.731
Xiangxi Prefecture	0.713	0.691	0.801	0.634	0.634	0.684	0.654
**Southwest China**							
**(Chongqing)**							
A[a]	0.577	0.543	0.597	0.587	0.614	0.659	0.532
B[b]	0.619	0.551	0.509	0.652	0.534	0.533	0.604
C[c]	0.697	0.742	0.575	0.476	0.568	0.664	0.613
D[d]	0.340	0.500	0.620	0.410	0.647	0.624	0.557
**Northern China**							
**(Jilin Province)**							
Changchun City	0.680	0.689	0.675	0.540	0.648	0.584	0.705
Jilin City	0.588	0.556	0.845	0.539	0.754	0.770	0.751
Siping City	0.733	0.851	0.560	0.704	0.529	0.544	0.665
Liaoyuan City	0.692	0.513	0.760	0.577	0.664	0.733	0.644
Tonghua City	0.517	0.581	0.703	0.618	0.657	0.662	0.628
Baishan City	0.535	0.411	0.878	0.760	0.710	0.894	0.624
Songyuan City	0.586	0.461	0.197	0.575	0.499	0.339	0.449
Baicheng City	0.737	0.391	0.766	0.642	0.830	0.698	0.521
Yanbian Prefecture	0.457	0.437	0.367	0.532	0.558	0.536	0.266

P=0.000

[a]: The central urban area of Chongqing; [b]: The new area of Chongqing city proper; [c]: The city cluster

of three gorges reservoir area in northeast Chongqing; [d]: The city cluster of Wuling mountain area in southeast Chongqing
